# The Antecedent Treatment of Those Exposed to Zymotic Diseases

**Published:** 1878-03

**Authors:** 


The Antecedent Treatment of Those Exposed to Zymotic Diseases.—An address before the American Medical Association (1877), and two articles on individual sanitation (*Medical Record*, N. Y., Nos. 359, 370), have brought me letters from individuals—some of whom are personally unknown by me—sufficient to show that the subject is one eliciting much inquiry. I am asked to state definitely the theory of prevention, and the details as applicable in practice.

First of all, let it be understood that the proposal to prevent or abate zymotic diseases by antecedent medication is based upon two views:

1. That the contagium vivum is introduced into the human system from without, and mostly through inbreathed air. The proposition is so to surcharge the mouth and nostrils with substances inimical to the lodgment, propagation or absorption of the contagium as to render the breath and the tissues more immediately exposed to inspiration unfriendly to the development of the contagium. When the floating infective particle presents itself, either for local manifestation or for absorption, it may take but slight unfriendliness of reception to prevent morbid result. I know not how little sulphur, or even lard alone, will prevent the egg deposit of the acarus scabiei or its incubation. The same is true as to the attachment of fungi, spores, etc. What we know as to both plants and animals shows how conditions of surface, and even odors, determine choice of location, and the "instinct" of "infectants" shows choice and interruption not less wonderful than that which occurs in animal and vegetable (parasitic) life. In diseases such as diphtheria and scarlet fever, which have, at the start, such marked local manifestations as to lead to the hypothesis that they are local before they are constitutional, there is indication for special localization of protective methods besides the constitutional prevention or treatment.

In an article on diphtheria in your journal of Jan. 23, 1875, we therefore insisted upon the value of local medication, although aware of the disposition of some good authorities to ignore it. Local applications to the membrane and its edges, local ablution of the fauces to prevent the adherence or absorption of infected particles, avoidance of swallowing the spittle or secretion, and frequent rinsing of the mouth, were advised. Our design and method is not to leave the mucous membrane of the air-passages without the presence of the antiseptics. We even value the muriatis ferri chlorid. the more because of its corrugal or mechanical effect, as it thus disturbs the apposition of surfaces. In an article on scarlet fever in the *Record*, June 19, 1875, the same fréquent cleansing of the throat, with a view to local effect, is insisted on. It somewhat surprises us to hear one of your recent contributors speak of his new method of frequent local medication.

Our second proposition is so to introduce substances into the blood, and through it into the body, as to prevent those fermentive, defibrinizing, or destructive processes which come to constitute the peril of these diseases. The article just alluded to sufficiently presents the method as adopted for early treatment. But we believe further that certain substances thus introduced into the blood, and mingling with the secretions before the prodroma are manifested, are still more operative for the prevention or limitation of the infective diseases. We thus not only protect the portals of entrance, but so infuse the fluids, glands, etc., as to have present in them an agent which will resist such morbid action as the disease toxic seeks to set up. How much may be due to the local hindrance or fertilization, and how much to the constitutional resistance established, may not, in each case, be easy to determine. But when we find how readily chlorate of potassium, after being administered, is found in the secretions, how soon a few grains of pure protochloride of iron increase by count the red globules of blood—so constant an occurrence in some of these diseases—is retarded by certain additions to the vital fluid, we are very hopeful that our power to suspend the action of disease poisons will be greatly augmented.

With quinine the experiment has been sufficiently sustained. It is taken—and taken successfully—as a prophylactic, by persons who have not had ague, but who are compelled to traverse miasmatic districts. The ratio of escape, and the value of such treatment to prevent recurrence, simply mean that somehow such resistance of the system is established as prevents the usual manifestation of morbid power. The miasm is inbreathed, but the receptivity of the system is suspended. We would experiment in the same direction in reference to all diseases, the causes of which enter by atmospheric conveyance. The principle, too, is applicable to some of the diseases conveyed by imbibition. It will be a great aim if, in addition to seeking and finding the *contagium vivum*, or the conditions of its propagation by external nuisances, we can also exercise upon the individual prophylaxis, which means "*to defend beforehand.*" The doctrine of the incompatibility of certain substances with the development of zymotic diseases, is quite in accord with views which have been entertained or illustrated by facts. The influence of the sulphites, as shown by Prof. Polli, of Milan, we have before noted. The interference of vaccination with the progress of whooping cough, has long been asserted by various reputable practitioners.

Dr. E. J. Syson, one of the English Medical Officers of Health, in a paper noticed in the *London Sanitary Record*, November 23, 1877, as of great importance, advocates the antagonistic treatment. He was led by some facts coming under his notice, "to infer that it was impossible to vaccinate successfully a patient who was at the time under the medicinal influence of arsenic." In other words, it is impossible to put the system in a condition temporarily unsusceptible to the "*contagium vivum.*" He applied this mostly to the use of arsenic as an antiseptic in cases of actual attack. So important do the facts seem to the reviewer, that he says, if they are corroborated by other trials, the value of such treatment will be but little less than that of the discovery of vaccination. The paper of Prof. Hogdèn, on Antiseptic Surgery (International Medical Congress, 1977) is worthy of reading in its bearing on antiseptic medicine. The third conclusion is, "It is possible that the living solids and liquids of the body may be so altered that they shall not furnish the conditions necessary to putrefaction." So, as to zymotic diseases, I believe we are yet to come to a recognition of our power by the use of medicines in advance to protect from prevalent epidemics. One class of agents may do it by the action of presence, another by chemical reaction; another by such antiseptic action as suspends excessive metamorphosis, or so deals with debris as to render it innocuous. Others, as is claimed for ammonia and liquor potassæ, retard the coagulation of fibrin. We are no longer confined to chemical antidotes, although these may operate upon disease toxics as well as others. Ergot and nitrate of amyl affect the calibre of blood-vessels, on a plan different from that of gallic acid.

Oxygen, electricity, ammonia, etc., are showing powers quite different from those of an alkali upon an acid. Just now we are not so concerned about the theory of action as about the fact. The need is to accumulate evidence by those whose trials are exact in the details of method and in the record of results. We cannot at present be decisive as to all the agents which interfere with zymotic infections, or as to those which are especially adapted to each particular form. We need only now indicate those upon which we have relied, and the method of their use. The articles themselves are not new, but they are claimed to be preventives, to be used before any manifestation of disease in the individual. We shall here merely outline the method of individual sanitation adopted in diphtheria and scarlet fever as a specimen of the general course of management.

In the case of all children in the family beside the one attacked, give the skin a washing or wiping each day with lukewarm carbolic wash of the strength of Squibb's 2 per cent. disinfectant solution of the mixed phenols. As we have always inclined to the view of a localized planting or absorption, we use frequent local treatment. A gargle of chlorate of potassium three grains, tinct. chloridi ferri three drops, water three drachms, is to be used frequently in the mouth, and some of the same is used about the nostrils. Frequency at first is far more important than quantity. Employ both what is used as gargle and for administration every two hours in the day, and every two or three hours between 10 P.M. and 6 A.M We give internally every three hours the same amount as named above for a gargle, and every two hours if there has already been more than two days of exposure. After one or two days the chlorate of potassium and the iron can be detected in the secretions. So the buccal cavity and the fauces have a share from this source, and the local use may be lessened one-half in frequency. The design is not to leave the mouth or the system without the indicated presence of these two articles, during what would be the prodromal stage of the disease. In diphtheral exposure we give in addition two grains of quinine twice, each six hours apart. It is important to have the tincture muriatis ferri of that made several months, and in its purest form, in which, and in which only, it contains the "sesquichloride of iron, muriatic acid, and alcohol, and from the reaction of the last two ingredients muriatic ether." "It is," says Wood, "possessed of peculiar properties, probably in some measure owing to the ether which it contains." In the preventive method for scarlet fever we do not use quinine. In addition to the diphtheral treatment we use Calvert's carbolic acid in a weak solution twice per day, and cleanse the teeth and mouth with a tooth-powder of equal parts of chlorate of potassium and powdered gum arabic. More attention is to be paid to a restriction of animal food with children who are full habited, and if the iron produces any constipation, as it generally does not, a mild cathartic is of service. Fuller particulars as to these or other diseases are not needed beyond those given in previous articles, and until more observations from others are accumulated. Reports either of failure or of success need to be explicit as to the length of medication and the precise frequency, and the whole amount of preventive used; also as to the degree of severity in cases in which attack is not avoided. Our position as to the plan of prevention is not that of dogmatism, but only that of an impressed inquirer. Sir Thomas Watson has ably written on the abolition of zymotic diseases, from the stand-point of outside sanitation. There is internal sanitation as well. We do not fasten our faith to any one remedy, but believe there is a field of observation in the area of personal disinfection and antiseptic provision which may yet show us how to invalidate the title and so take possession as to dispute the occupancy of many an infeetive disease. It is not by large dosing or inadvertent medication, but by that intelligent supervision and timely contravention which make the ounce of prevention more potent than a pound expended for attempted cure—too often failing just because the vicious poison has already so affected fluids and functions and organs as to impede those processes which are not less indispensable for the physiological effect of medicines, than for that of all vital repair.

If "antiseptics may alter the tissues of a wounded surface so that, though septic matters are present, they may not be absorbed," and if living solids and liquids may be so altered that they shall not furnish the conditions necessary to the development of disease, we will not despair of "prohibiting," of "having beforehand" in the system unsusceptibility to the invaders, such as will not respond to their attempt at sovereignty.—*Medical Record.*

